# An isolate of *Vibrio campbellii* carrying the *pir*^*VP*^ gene causes acute hepatopancreatic necrosis disease

**DOI:** 10.1038/emi.2016.131

**Published:** 2017-01-04

**Authors:** Xuan Dong, Hailiang Wang, Guosi Xie, Peizhuo Zou, Chengcheng Guo, Yan Liang, Jie Huang

**Affiliations:** 1 Key Laboratory of Sustainable Development of Marine Fisheries, Ministry of Agriculture, Yellow Sea Fisheries Research Institute, Chinese Academy of Fishery Sciences, Qingdao 266071, China; 2 Laboratory for Marine Fisheries Science and Food Production Processes, Qingdao National Laboratory for Marine Science and Technology, Qingdao 266071, China

**Dear Editor,**

In recent years, acute hepatopancreatic necrosis disease (AHPND) has rapidly spread in Asian countries and Mexico, causing severe mortality (up to 100%) and decreasing shrimp production.^1, 2, 3, 4, 5, 6, 7, 8, 9^ AHPND was originally shown to be caused by a specific virulent strain of *Vibrio parahaemolyticus*, namely the AHPND-causing *V. parahaemolyticus* (VP_AHPND_).^1, 5, 6^ *V. parahaemolyticus* becomes virulent VP_AHPND_ after acquiring a plasmid (pVA1) expressing the deadly toxin Pir^VP^, which consists of two subunits, PirA and PirB, and is homologous to the Pir (Photorhabdus insect-related) binary toxin.^7^ The plasmid pVA1 also carries a cluster of genes related to conjugative transfer; hence, this plasmid may potentially be able to transfer not only among *V. parahaemolyticus* strains but also to different bacterial species.^7, 10^ So far, there have been no published reports directly demonstrating that *Vibrio campbellii* can harbor *pir*^*VP*^ and cause AHPND in shrimp. In this paper, we challenged *Litopenaeus vannamei* with a strain of *V. campbellii* (20130629003S01) carrying *pir*^*VP*^ isolated from a *L. vannamei* farm and demonstrated that *V. campbellii* is a causative agent of AHPND.

In this paper, strain 20130629003S01 was isolated in June of 2013 from diseased *L. vannamei* in Guangxi, China. PCR and RT-PCR amplifications were performed using VpPirA and VpPirB primers specific to *pir*^*VP*^ genes (*pirA* and *pirB*).^11^ The electrophoresis of PCR products showed that both *pirA* (284 bp) and *pirB* (392 bp) were detected in the strain (Figure 1A). A partial sequence of 16S rRNA was obtained by sequencing the PCR products obtained with primers 27F (5′-AGA GTT TGA TCC TGG CTC AG-3′) and 1492R (5′-TAC GGC TAC CTT GTT ACG ACT T-3′).^12^ We found that strain 20130629003S01 belongs to the *Vibrio* core group, and its closest relatives are *V. campbellii* (99.72%) and *V. rotiferianus* (99.66%), according to the EzTaxon server (www.eztaxon-e.ezbiocloud.net). Partial sequences of σ^70^ factor (*rpoD*), replication initiator protein (*rctB*) and toxin transcriptional activator (*toxR*) were amplified as described by Pascual *et al.*^13^ After alignment of sequences for 16S rRNA and the *rpoD*, *rctB* and *toxR* genes, the phylogenetic tree was constructed with concatenated sequences by using neighbor-joining analysis in MEGA 5 (Tempe, AZ, USA). The multilocus sequence analysis clearly identified strain 20130629003S01 as being the closest to *V. campbellii* (Figure 1B).

Significantly, *pir*^*VP*^ genes were successfully amplified by using plasmid DNA extracted from the *V. campbellii* isolate (20130629003S01). In addition, next-generation sequencing of strain 20130629003S01 demonstrated that it also contains a pVA1-like plasmid containing *pir*^*VP*^ (unpublished data). Protein profiles of the crude protein fractions of strain 20130629003S01 were analyzed as described by Sirikharin *et al.*^14^ Analysis by SDS–polyacrylamide gel electrophoresis revealed two target bands at marker levels of ~17 kDa (PirA) and 50 kDa (PirB; Figure 1C). Mass spectrometry analysis followed by the MASCOT analysis revealed that the two proteins had similarity to the published protein sequences of PirA (gi|922664586) and PirB (gi|922664588) of *V. parahaemolyticus* M0605.

The pathogenicity of strain 20130629003S01 was examined in healthy *L. vannamei* shrimps weighing ∼1 g, which were reared in 90 l artificial seawater at salinity 30 in plastic tanks (density 15 shrimps/tank) at 27±2 °C. An immersion challenge was used to follow the bioassay protocol described by Tran *et al.*^5^ All experimental groups were assayed in triplicate. Shrimp immersed with the bacterial suspension began to develop typical gross signs of AHPND within 12 h, massive mortalities occurred from 12 h post challenge, and cumulative mortalities reached 100% within 36 h. Gross signs of challenged *L. vannamei* included an empty stomach and gastrointestinal tract as well as pale and atrophied hepatopancreas (Figure 1D). A histopathological examination of moribund shrimp revealed the presence of AHPND lesions (Figure 1E) characterized by the acute sloughing of hepatopancreatic tubule epithelial cells, some of which displayed intact organelles, such as nuclei and cytoplasmic vesicles (Figure 1E). To our knowledge, our study is the first to demonstrate that a *V. campbellii* strain carrying *pir*^*VP*^ causes AHPND. Therefore, AHPND caused by non-*V. parahaemolyticus* should be further investigated.

The shrimp farming industry is one of the important economic industries for countries in Asia and Latin America. AHPND is characterized by the acute and massive mortality in shrimp farms, causing severe production collapses and heavy economic losses. Ignoring the biosecurity of shrimp hatcheries and farms provides possibilities for the spread of VP_AHPND_. The existence of *pir*^*VP*^ in non-*V. parahaemolyticus* isolates has been reported in a *Vibrio harveyi*-like stain from Vietnam^15^ and a *Vibrio owensii*-like from China.^10^ The present results may provide evidence for the horizontal transfer of the *pir*^*VP*^ gene or pVA1 plasmid between different bacterial species, thereby potentially increasing the complexity of causative agents of AHPND and aggravating the threat to the shrimp industry. On the basis of our finding that a *V. campbellii* carrying *pir*^*VP*^ causes AHPND, effective biosecurity measures should be considered to prevent the spread of AHPND in the future.

## Figures and Tables

**Figure 1:**
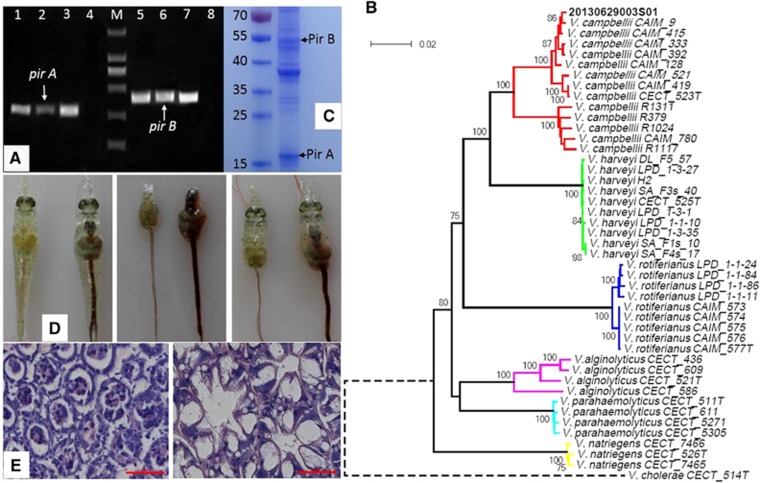
Identification and challenge tests of an isolate of *Vibrio campbellii* carrying *pir*^*VP*^ genes associated with AHPND in *Litopenaeus vannamei.* (**A**) Detection of *pirA* and *pirB* genes. Lanes 1 and 5: PCR results from total DNA of strain 20130629003S01; lanes 2 and 6: RT-PCR results from extracted RNA of strain 20130629003S01; lanes 3 and 7: PCR results from purified plasmid DNA of strain 20130629003S01; lanes 4 and 8: non-template control; lane M: DL2000 DNA marker. (**B**) Phylogenetic reconstruction based on concatenated 16S rRNA, *rpoD*, *rctB* and *toxR* sequences. Percentage bootstrap values (1000 replicates) >75% are shown. Bar, 0.02 expected nucleotide substitutions per site. The reference sequences were as described by Pascual *et al.*^13^ The nucleotide sequences from strain 20130629003S01 have been submitted to the GenBank database under accession number KX534746 (16S rRNA), KX534747 (*rpoD*), KX534748 (*rctB*) and KX534749 (*toxR*). (**C**) SDS–polyacrylamide gel electrophoresis analysis of PirA and PirB from broth of strain 20130629003S01. (**D**) Gross signs of AHPND-infected shrimp (left): pale, atrophied hepatopancreas, empty stomach and gastrointestinal tract. Normal shrimp (right) in the negative control group: normal size hepatopancreas with brownish color and full stomach and gastrointestinal tract. (**E**) Hematoxylin and eosin-stained histological sections of the hepatopancreas of *Litopenaeus vannamei* from challenge tests. (Left) AHPND pathology characterized by sloughing of hepatopancreatic tubule epithelial cells. (Right) normal shrimp hepatopancreatic histology. Scale bars=50 μm. Abbreviation: acute hepatopancreatic necrosis disease, AHPND.
